# Endocrine Disruptors and Pregnancy: Knowledge, Attitudes and Prevention Behaviors of French Women

**DOI:** 10.3390/ijerph14091021

**Published:** 2017-09-06

**Authors:** Steeve Rouillon, Chloé Deshayes-Morgand, Line Enjalbert, Sylvie Rabouan, Jean-Benoit Hardouin, Group DisProSE, Virginie Migeot, Marion Albouy-Llaty

**Affiliations:** 1 INSERM, University Hospital of Poitiers, University of Poitiers, Clinical Investigation Center 1402, 2 rue de la Milétrie, 86021 Poitiers CEDEX, France; steeve.rouillon@univ-poitiers.fr (S.R.); chloe.deshayes.morgand@etu.univ-poitiers.fr (C.D.-M.); sylvie.rabouan@univ-poitiers.fr (S.Ra.); virginie.migeot@univ-poitiers.fr (V.M.); 2 Faculty of Medicine and Pharmacy, University of Poitiers, 6 rue de la Milétrie, 86000 Poitiers, France; 3 Department of Public Health, BioSPharm Pole, University Hospital of Poitiers, 2 rue de la Milétrie, 86021 Poitiers CEDEX, France; marion.albouy-llaty@chu-poitiers.fr; 4 UMR CNRS 7285, IC2MP, 86073 Poitiers CEDEX 9, France; 5 INSERM U1246, University of Nantes, 44035 Nantes, France; line.enjalbert@etu.univ-nantes.fr (L.E.); jean-benoit.hardouin@univ-nantes.fr (J.-B.H.)

**Keywords:** endocrine disruptors, risk perception, exposure reduction, pregnancy

## Abstract

Endocrine disrupting chemicals (EDC) are environmental exposure factors that are rarely reported in clinical practice, particularly during pregnancy. This study aimed to describe women’s knowledge, attitudes and behaviors towards EDC exposure. A study was conducted in the French Department of Vienne between 2014 and 2016 and comprised semi-structured interviews with pregnant women, a focus group of professionals in perinatology and environmental health, and the administration of a psychosocial questionnaire comprising scores in 300 pregnant or in postpartum period women. The mean score of knowledge was 42.9 ± 9.8 out of 100 (from 13.5 to 75.7). Exposure attitude was determined by risk perception. Mean level of cues to action to reduce their EDC exposure was estimated at 56.9 ± 22.5 out of 100 (from 0 to 100). Anxiety was significantly increased after the questionnaire. Anxiety about EDC was associated with a high score of knowledge (OR = 2.30, 95% CI (1.12–4.71)) and with no pregnancy anxiety (OR = 0.57, 95% CI (0.34–0.95)). Our findings suggest that healthcare providers should consider pregnant women’s knowledge and perceptions, possibilities of action, and be careful not to increase their anxiety when advising them about EDC and environmental exposure.

## 1. Introduction

According to the “Developmental Origin of Health and Disease” hypothesis (DOHaD hypothesis), fetuses are particularly susceptible to the impact of nutritional and environmental factors during the in utero period, with long-term health consequences in childhood and adulthood [1]. Some of these environmental factors are endocrine disrupting chemicals (EDC). EDC are natural or synthetic chemical molecules able to modify an organism’s operation of the hormonal system [2].

EDC come from different categories, for example plastics like bisphenol A (BPA), pesticides or personal care products (i.e., parabens) [3]. EDC have been found in maternal and cord blood [4], urine of pregnant women [5] and colostrum [6]. Because of the trans-placental transfer of these molecules, fetuses are particularly exposed [7].

Many diseases and disorders are considered as being related to prenatal exposure to EDC, including fetal development disorders associated with low birth weight [8], obesity [9,10,11], prematurity [12], autism [13], allergies [14], and pubescent development disorders [15]. Cancers are also a possible consequence of EDC exposure [16].

Being aware of the state of vulnerability of pregnancy, the French medical profession provides advice for pregnant women, but not always about EDC [17,18]. However, due to the ubiquity of EDC in our environment, gynecologists have begun to get involved [19]. Moreover, some recommendations about EDC for pregnant women, originating in worldwide studies, are now being suggested [20,21,22]. “Do not color your hair and use as few cosmetics and lotions as possible during pregnancy”, and “avoid the use of some kinds of plastics and reduce consumption of canned foods”; are but a few examples. Such recommendations or advice may be customized and therefore, take a pregnant women’s perception of EDC into account, as is already suggested for other subjects (e.g., dietary and physical activity during pregnancy [23]). Moreover, the development of pregnant women’s abilities to make their own healthy choices is now encouraged, by the use of leaflets or the creation of educational programs on environmental health [24].

Mainly through the use of cosmetics or personal care products, recent studies begun to assess behavior of pregnant women towards EDC and chemical compounds [25], and the risk which may result to a chronic use of these products [26,27]. Aims of this study were to describe women’s knowledge, attitudes and behaviors towards EDC, explore EDC risk perception in this population, and assess the anxiety following hetero-administration of a pregnancy questionnaire about EDC.

## 2. Materials and Methods

### 2.1. Framework

**Figure 1:**
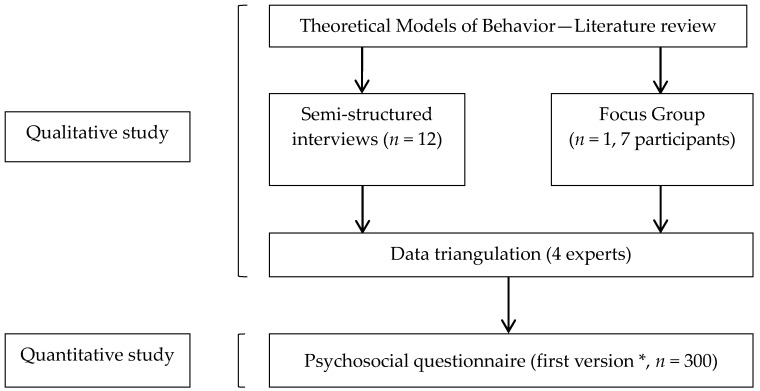
Main steps of the study. * This part of the study deals with the validation step of the psychosocial questionnaire. An adjustment step was led later on 30 women.

Our study was composed of three main steps: a qualitative study, with semi-structured interviews of pregnant women, a focus group of professionals, and a quantitative study administering a psychosocial questionnaire to pregnant or postpartum period women. A psychosocial questionnaire related to health behaviors, as our questionnaire, assesses the relations between individual psychological variables and variables related to the social context in order to better describe health behaviors in front of EDC risk exposure. These steps are detailed in Figure 1.

We chose a theoretical model of health behavior revolving around risk perception: the Health Belief Model (HBM). In this model, severity, susceptibility, benefits, barriers, and cues to action determine probability of health behavior change [28]. These criteria were used to build the interview grids for the semi-structured interviews and for the focus group, which were used then to design the psychosocial questionnaire in two main parts: (i) a first part assessing risk perception, with severity and susceptibility to EDC risk exposure and (ii) a second part assessing benefits, barriers and cues to action to adopt a healthy behavior.

The first step of the study (semi-structured interviews) was performed in 2014 in the city of Poitiers (France) by a student midwife. This consists of the use of a topic guide that contains open-ended question and provides a great flexibility to explore experiences and attitudes. A semi-structured interview provides much information. The target population was composed of adult pregnant women, speaking French, who had previously had children or not, and were consulting for pregnancy monitoring at the University Hospital of Poitiers or in a private midwifery office in Poitiers. From medical records, a panel was formed taking age and type of housing into account in order to recruit a diversified range of pregnant women. The panel for semi-structured interviews was composed of 12 pregnant women. Socio-demographic data on pregnant women are detailed in Appendix A, Table A1. The semi-structured interviews were recorded once in an audio file. They were manually transcribed afterwards. The length of each interview was about one hour. All data were processed anonymously. Verbatim were not given to the women and idea saturation was sought out. The interviews took place in a confidential area. The partner or a friend of the pregnant woman was sometimes present.

The second step was the focus group of professionals. It took place in March 2015 on the premises of the Faculty of Medicine and Pharmacy of Poitiers. The target population was composed of professionals from different fields in perinatology, health promotion and environmental health education. The professionals, or future professionals, were a student midwife, a pediatric nurse from a French departmental structure responsible for mothers and their children’s protection, a student in prevention psychology, a project leader at the French health care mutual, a project leader at a French association involved in health education and promotion, an organizer of health education workshops and a Ph.D. student in environmental health. Their characteristics are detailed in Appendix A, Table A2. These seven professionals did not know each other before the focus group and had no marked hierarchical links in order to ease each person’s speech, which was completely free. Three main questions were posed to this group, as relaunches, during the focus group: (i) “How you would talk about perinatal exposure to endocrine disruptors?”; (ii) “What factors are likely to interfere with the perception of exposure at the time of the interview with a woman, pregnant or not?”; and (iii) “What factors are likely to influence a change in behavior of this exposure to endocrine disruptors?”. The focus group lasted 90 min. It was recorded in the presence of an organizer (M.A.-L.) and an observer (J.A.) who was asked to note the physical language of every participant. Idea saturation was searched, until no new information was brought forth, according to focus group methodology. While a private midwife was not able to join the focus group, her ideas were collected during a semi-structured individual interview.

In the qualitative study, analysis of the semi-structured interviews and focus group was processed by examination of the verbatim, in three phases: (i) extraction of all information, (ii) detection of the relevant data and (iii) organization in logic trees. The themes were not identified in advance. The analytical “triangulation” method was chosen. Data were selected and sorted out using the RQDA qualitative analysis software, a CAQDAS-type software (Computer-Assisted Qualitative Data Analysis Software) running on the [R] program (R development core team).

Then, using the information gained from the qualitative study, we constructed a questionnaire to assess women’s knowledge toward EDC, attitudes such as EDC risk perception and anxiety, and behaviors to reduce EDC exposure. It comprised 37 questions divided into 4 sections.

The third step consisted in the administration of this questionnaire to pregnant women or in postpartum period.

### 2.2. Population and Recruitment

A cross-sectional study was performed between 18 August 2015 and 8 April 2016 in French department of Vienne. Women were informed of the study by clinicians, leaflets in participant midwives’ offices (in and around the city of Poitiers) and in the 3 maternity units of the department, and on a social network.

Eligible subjects were pregnant women with a singleton pregnancy without complication, or women having given birth and being hospitalized with their healthy newborn in a maternity unit with a vaginal or uncomplicated cesarean delivery, French-speaking and aged 18 or older.

Before each interview, a simple explanation was given concerning the theme of the study. All women gave written informed consent. This study was approved by the local ethics committee (Comité de Protection des Personnes Ouest III, reference 2015-A00031-48, date of approval: 19 May 2015).

### 2.3. Data Collection

For the cross-sectional study, data were collected by a questionnaire in an interview with a researcher in the hospital room for women in postpartum period or in a medical office for pregnant women. The researchers were trained to limit information bias. This questionnaire contained visual analog scales which were scored from 0 at the left extremity to 100 at the right extremity.

Socio-demographic data (age, profession, education level, marital status and parity) and smoking status were collected in medical records.

#### 2.3.1. Knowledge

We explored women’s knowledge about EDC, with questions about definition, ability to give some names, source of exposure, way of exposure and knowledge about how to avoid EDC. These items were assessed with closed-questions, except for the knowledge of molecules’ names. That allowed us to construct an EDC knowledge score with a maximum of 100 points. We used photo-language^®^ to increase the accessibility of the questions on exposure sources, knowledge of plastic packaging resin identification codes and those to avoid in daily life [20,29,30]. There was also a question on perceived knowledge about EDC assessed with a visual analog scale. After this part, EDC definition such as “chemical mixtures in the environment that possess properties to alter function(s) of the endocrine system” was given to the women.

#### 2.3.2. Attitude: Perception of EDC Risk

Perception of EDC risk for both maternal health and fetal health was then explored in the questionnaire. Questions like “EDC risk for my health is” or “EDC risk for my baby to have a low birth weight is”, or also “EDC risk for my baby to have fertility trouble in adulthood is” were assessed by the women on a visual analog scale and in a general way in three grades: null, low or high. Risk assessment “in a general way” relates to *perceived severity*, whereas risk assessment for a given pregnant woman or a given child is considered as *perceived susceptibility*. This part ended by assessing the concept of what a healthy baby is: women were asked to agree or disagree with several statements, for example “a healthy baby has normal weight at birth” [31,32]. Moreover, the hierarchy of risk during pregnancy between genetic and metabolic diseases, infectious diseases, toxic diseases, chemical-related diseases and pregnancy ailments was a subject on which the participants were interrogated.

#### 2.3.3. Behaviors

We evaluated behaviors through cues to action. There were open questions on possible actions to limit EDC exposure like “how do you think you can act?” and questions to assess the efforts to reduce exposure with a visual analog scale.

#### 2.3.4. Anxiety

Women evaluated their own situational anxiety before and after answering the questionnaire on a visual analog scale: the left extremity was for “Not anxious” and the right extremity for “Very anxious”. Anxiety in the preceding days and general anxiety were also assessed with the same tool. This approach of measuring both situational and general anxiety trait was inspired by the State-Trait Anxiety Inventory [33,34].

### 2.4. Statistical Analysis

In the cross-sectional study, continuous variables were expressed as mean, standard deviation (SD) and quartile. Categorical variables were expressed as frequency and percentage. The difference of situational anxiety between after and before administering the questionnaire was used to define the change of anxiety due to the administration of the questionnaire. A paired *t*-test was performed to assess change in anxiety when answering the questionnaire. The change in anxiety was then categorized as “increased anxiety” if the difference was strictly greater than zero point, or “stabilized or decreased anxiety” if this difference was equal to or less than zero point. Continuous variables such as perceived health, general anxiety and knowledge about EDC score were then categorized in quartile according to sample distribution. Bivariate analyses were performed to assess anxiety increasing with factors as age, socio-professional category, perceived health, general anxiety, pregnancy anxiety and knowledge about EDC. A multivariate logistic regression model was applied to assess predictors of increased anxiety. Variables that were associated with anxiety increasing at a *p-*value of <0.20 in bivariate analysis were included in the model except for age. All analysis was conducted in SAS 9.4 (SAS institute Inc., Cary, NC, USA) and Stata Statistical Software: Release 14 (College Station, TX, USA: StataCorp LP).

## 3. Results

### 3.1. Women’s Characteristics

**Figure A1:**
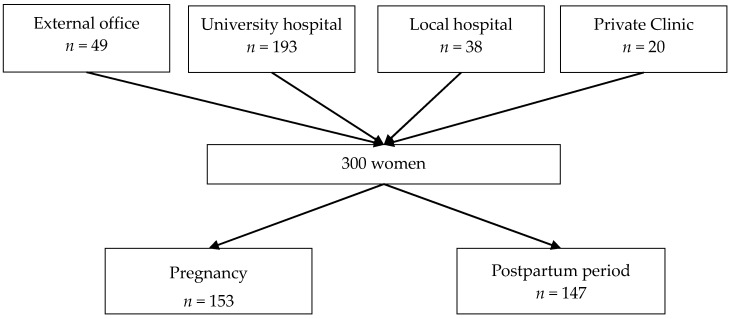
Flowchart of the quantitative study population.

Three hundred women were included in the study. Their mean age was 30.9 ± 4.7 years and they had 1.2 ± 1.0 children (Table 1). Sixty-four percent of women were cared for by university hospital, 12.7% by a local hospital maternity, 6.7% by a private clinic and 16.3% by a midwife in an external office (see flow chart in Appendix A, Figure A1). More than half (51%) were pregnant women and 49% were women in postpartum period. Women had mainly (77.0%) a university education level. The mean of perceived health was 80.7 ± 17.6 out of 100.

### 3.2. Knowledge

In this sample, 54.3% of women had never heard about EDC. The mean score of knowledge of ED was 42.9 ± 9.8 out of 100 (from 13.5 to 75.7). The mean score of perceived knowledge about EDC was 19.0 ± 16.6 out of 100 (from 0.0 to 78.0) (Table 2).

An EDC was mostly defined as a molecule altering the functioning of the body (83.3%). Sources of EDC exposure most widely named were cosmetics (91.3%). Plastic packaging resin identification codes were unknown by 50.7% of women.

Among the 137 women who had heard about EDC, the EDC cited were primarily pesticides (26.3%), bisphenol A (25.6%) and parabens (24.1%). The average number of EDC named was 0.9 ± 1 (from 0 to 4). Main vectors of information were media (64.2% television, 46.0% magazine, and 38.7% Internet), friends (47.5%), work (37.2%) and health professionals (4.3%). These women felt that information was understandable (80.3%), complex (68.6%), alarmist (67.9%), stressful (56.9%), or overly scientific (33.6%).

### 3.3. Attitude: Perception of EDC Risk

**Figure A2:**
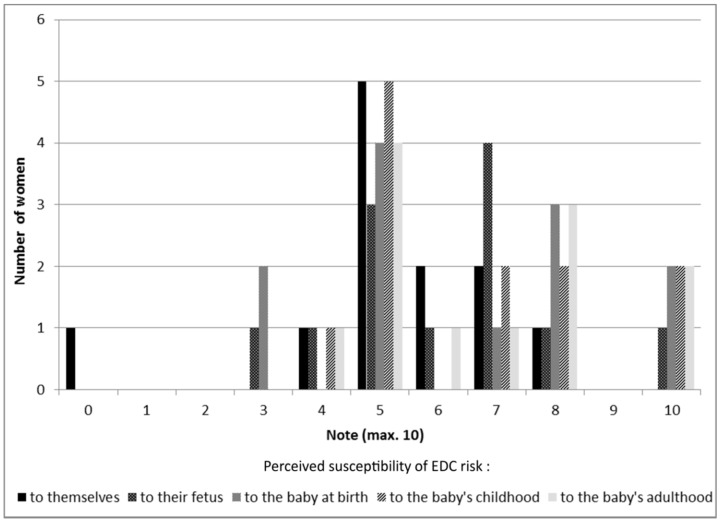
Distribution of pregnant women’s perceived susceptibility to endocrine disrupting chemicals (EDC) risk exposure (qualitative study, *n* = 12).

From pregnant women interviews, risk perception, particularly *perceived susceptibility*, changed with the target (the pregnant woman, her fetus, the future newborn, teenager and adult). Distribution of pregnant women’s answers to the question “Do you think there is a risk related to exposure to these chemical molecules for yourself? And for your baby? On a scale from 0 (no risk) to 10 (maximal risk)” is represented in Appendix A, Figure A2. Median notes suggested that women were more perceptive to the risk related to EDC exposure for their child than for themselves (data not shown).

Risk assessment for the women and their children is detailed on Table 3. All women reported general risk related to EDC for women’s health but between 3.0% and 25.3% of women considered risk to be null for children health depending on the health issue.

Chemical-related diseases were in fourth position in hierarchy of risk during pregnancy with a score of 72.1 ± 27.7 out of 100 after genetic and metabolic diseases (78.1 ± 24.7 out of 100), infectious diseases (74.4 ± 29.1 out of 100) and toxic diseases (72.5 ± 34.7 out of 100) (data not shown).

### 3.4. Behaviors

Among the 300 women, mean level of cues to action to reduce their EDC exposure was estimated at 56.9 ± 22.5 out of 100 (from 0 to 100). Women suggested a need to check labels and recycling codes (44.0%), to consume products of organic farming (35.0%), to consume fresh products (31.0%), to reduce consumption of industrial products (26.0%), to consume products from their gardens (23.0%), to use glass containers (21.0%), to reduce use of plastic containers (18.7%), to limit household chemicals (18.0%), to limit consumption of food packaged in cans (14.7%), or to reduce the use of cosmetics (13.3%).

One hundred twenty-one women (40.3%) already used or intended to use chemical-free products during pregnancy and 107 found a solution: 73 (68.2%) were inclined to reduce consumption of industrial products, 67 (62.6%) to use glass containers, 62 (57.9%) to reduce plastic container use, 62 (57.9%) to not heat food in plastic containers with a microwave oven and 61 (57.0%) to reduce consumption of canned food.

The majority of women (92.0%) were ready to change their habits to avoid exposure, but 86.7% considered their habits to be of major importance. Efforts to avoid chemical exposure were estimated at 57.9 ± 20.7 out of 100 (from 0 to 100) for financial efforts, at 55.1 ± 23.9 out of 100 (from 0 to 100) for efforts in terms of time, and at 52.5 ± 24.4 out of 100 (from 0 to 100) for efforts in terms of comfort and, respectively, 44 women (14.7%), 20 (6.7%) and 31 (10.4%) of them were not ready to make these efforts.

One hundred eighty-nine women were inclined (63.0%) to make their own consumer products: 42.3% prepared their meals, 28.0% their desserts, 19.0% their bread and 17.0% their yogurt.

Among the 90 women (30.0%) who neither bought nor wished to buy products from organic farms, the most frequently mentioned reasons were price (48.9%), mistrust in the label (11.3%), low selection (10.0%), habits (7.0%) and accessibility (5.6%).

### 3.5. Anxiety about EDC Risk

In the cross-sectional study, general anxiety was assessed at 32.6 ± 24.3 out of 100 (from 0 to 100) and 53.7% of women reported increasing anxiety during pregnancy. Mean situational anxiety levels before and after questionnaire were respectively 19.6 ± 19.8 and 27.3 ± 22.2 (Table 4), and the increase was significant (*p* < 0.0001). This was found in all classes of each variable except for the fourth quartile of perceived health and for the second quartile of the knowledge score of EDC. One hundred eighty-six women (62.0%) showed increased anxiety. In bivariate analysis, the probability of increasing anxiety was significantly associated with the fourth quartile of knowledge score (OR = 2.15, 95% CI (1.10–4.17)). Age, perceived health and increased general anxiety during pregnancy were not significantly associated with anxiety about EDC. Moreover, the difference of increasing anxiety was not significant between women who had increased anxiety during pregnancy and those who had not (data not shown). After adjustment on age, perceived health and parity, probability of increasing anxiety with the questionnaire was significantly associated with the fourth quartile of knowledge score (OR = 2.30, 95% CI (1.12–4.71)) and with increased anxiety during pregnancy (OR = 0.57, 95% CI (0.34–0.95)).

## 4. Discussion

### 4.1. Knowledge

This study illustrates the fact that women do not know much about EDC and potential sources of exposure. Moreover, women estimated they had a weak knowledge about EDC. That is concordant with a French local survey on environmental health where 47.4% of subjects interrogated in the general population had not heard about EDC and 68.8% felt that they did not know about their effect on health [35]. It is necessary to inform women about EDC, especially since they want to know its health effects, and consequently make informed choices [36,37]. The most widely named molecules were Pesticides, Bisphenol A and Parabens, which is concordant with a French study [38]. Bisphenol A is probably largely known because it is banned in baby bottles in France and pesticides are the subject of the greatest concern in environmental health [35].

There are many sources of information about EDC, and women may be overwhelmed by its amount and may perceive varying in quality and accuracy. However, pregnant women take into consideration the value of the source of information before possibly taking action: for example, although media are major vectors of information, they are perceived by women as weak sources [39]. While health professionals are considered as strong sources of information, only 4.3% of women were informed by them about EDC. This can be explained by the lack of information, training and scientific evidence in environmental health mentioned by them in other studies [26,36]. Perinatal health professionals have an important role in protecting pregnant women from chemical exposure because pregnancy is a susceptible period for exposure to EDC, and a majority of persons consider health professionals to be in the best position to answer their questions about environmental health or believe that it is their responsibility to inform them about EDC exposure [35,36,37]. However, information about environmental exposure prevention exists [19,20], even though is not addressed in the official recommendations.

Health authorities and the government could also serve as a vector, especially since they are considered as strong sources of information [39]. Leaflets to limit exposure to chemical exposure during pregnancy could be given to pregnant women [22].

### 4.2. Attitude: Perception of EDC Risk

The perception of the risk is a subjective assessment of the probability that a specific type of accident may occur; it shows to what extent the concerned individual estimates the consequences.

In this study, 70.9% of women considered EDC risk as high. That percentage is more elevated than in a recent French study where it was found that 50.4% of the women felt concerned by EDC risk for health [38]. No women reported no risk, which shows that EDC risk exposure was indeed always perceived in our study population.

Through the qualitative study, we identified age and socio-economic category as factor likely both increase and decrease the perception of the risk, depending on the situation. These results should be confirmed by quantitative analyses.

### 4.3. Behaviors

In this study, the majority of women were ready to change their habits to avoid exposure. Almost half of them proposed at least one action to reduce exposure to chemicals. The most widely cited proposal consisted in checking labels, suggesting that women are aware of the ubiquity of the EDC. Necessary effort in terms of cost, time and comfort was estimated at slightly superior to 50/100 and only a few women were not ready to act accordingly. Cost was identified in some studies as a limited factor [31,37]. Taking these barriers into account is important inasmuch as they can influence a woman’s decision. We found that 40% of women already use or intend to use chemical-free products during pregnancy. This change of practice may be associated with the EDC risk perception; a previous study showed that women who consider environmental chemicals as dangerous were more likely to have healthy behaviors [27]. A similar finding has been reported about exposure to tobacco during pregnancy; it has indeed been found that perceived fetal health risk was a predictor of anti-smoking behaviors of pregnant women [40]. This is also concordant with Ashley et al., who found that pregnant women adopt behaviors aimed at reducing EDC exposure after considering factors such as financial cost, legitimacy of the exposure risk and practicality [39].

In this study, the use of glass containers was proposed by almost a quarter of women. In a recent French study including women of childbearing age, the women who cited plastic as a source of exposure used plastic containers as much as women who did not cite it [38] which suggests that it is not because people are aware of the risk that they take action to avoid it. The same findings apply to cosmetics and personal care products, only 13.0% proposed to reduce cosmetic use, even though 91.3% agreed that they are sources of exposure to EDC. This finding shows that women realize that cosmetics use may be dangerous but probably do not know the risks of using cosmetics during pregnancy and consequently, as was found recently, continue to use them [26].

### 4.4. Anxiety from ED Questionnaire

In this study, more than a half of the participating women saw their situational anxiety increase as they answered the questionnaire about EDC knowledge, attitudes and behaviors. After adjustment on age, perceived health and parity, the probability of increasing anxiety was associated with better knowledge of EDC and increased general anxiety during pregnancy. This finding may be explained by the fact that women with more knowledge about EDC also have more knowledge about the risks associated with exposure. Barrett et al. found that educated women were more likely to believe that environmental chemicals are dangerous [27]. Moreover, EDC are one of the main subjects of concern in conversations about environmental health [35].

The probability of increasing situational anxiety along with information about EDC was also associated with no increased general anxiety during pregnancy. We found that situational anxiety increases significantly between before and after answering the questionnaire in both groups (“more” and “not more anxious” during pregnancy), but that the difference between these two groups was not significant. We suppose that this finding may be due to a kind of anxiety saturation: women who were already very anxious during pregnancy because of other risks may reach saturation whereas women who were not more anxious can have a greater margin of increase. This hypothesis needs to be confirmed by another study.

However, increased anxiety in women who are not more anxious during their pregnancy was not expected and illustrates the difficulty of informing women about EDC without increasing their anxiety. It is therefore important to take women’s knowledge and representations about EDC into account before providing information on EDC in perinatal care.

### 4.5. Strengths and Limitations of the Study

Since a student midwife conducted the semi-structured interviews, pregnant women were able to identify her as a health professional. This may have led to information bias because of reluctance to candidly express their thoughts and the fear of being judged. This bias, related to a certain social desirability, was also suspected on account of some contradictory answers found within the same interview. However, qualitative studies using semi-structured interviews have proven their efficiency [31,41], and pregnant women are more prone to engage with midwives than with other perinatal professionals [42].

Considering the participants of the focus group, we lacked an obstetrician-gynecologist. Since EDC are seldom approached in their medical practice today [19], as only 20% reported routinely asking about environmental exposures in pregnant women [18], and since the focus group is based on professional experience, it is possible that the presence of such a professional would not have brought new elements. Furthermore, the multi-disciplinary composition of the focus group enabled idea saturation.

The target populations were composed of pregnant women and perinatal and environmental health professionals. Indeed, the global perception of the risks and the associated judgments depended on the socio-economic context and the feeling of belonging to the same group [43]. Considering these cultural considerations, relevant to both pregnant women and professionals, the results of this study can be extended only to target populations presenting the same cultural and socio-economic characteristics.

This cross-sectional study presents some limitations. It may have a selection bias because its participants were volunteers, and we did not compare the women who participated in this study with those who did not wish to participate. Generalizing these results to the French population should be undertaken with the utmost caution. However, it was a multicenter study involving all maternity units in the department.

While many researchers conducted interviews of women, we intended to limit information bias by the researchers’ training and giving them a guide for investigator, so that the course of the interview was the same for all the women.

## 5. Conclusions

Our study provides important information on: (i) women’s low knowledge about sources of exposure and risks related to exposure to EDC; (ii) attitudes with EDC risk perception; (iii) women’s behaviors; and (iv) the anxiety generated by EDC.

Communication on this public health subject is likely to increase women’s situational anxiety. Our findings should induce health care providers to advise women about EDC and environmental exposure, based on their knowledge and representations about EDC, taking their cues to action into consideration, and taking care to avoid increasing their anxiety.

## Figures and Tables

**Table 1:** Women’s characteristics.

Characteristics		*N*	%
Status			
	Pregnancy	153	51.0
	Postpartum period	147	49.0
Age (years old)			
	Mean ± SD (min–max)	30.9 ± 4.7	(20.5–44.1)
	18–25	33	11.0
	26–35	206	68.7
	>35	61	20.3
Cared for by			
	University hospital	193	64.3
	Local hospital	38	12.7
	Private clinic	20	6.7
	External office	49	16.3
Educational level *			
	Elementary, secondary school	29	10.4
	High school	35	12.6
	University level	214	77.0
Marital status *			
	Married or with a committed partner	272	93.2
	Single	20	6.8
Socio-professional category *			
	Farmers, Artisans, entrepreneurs, workers, other	59	20.6
	Executive and intellectual professions	42	14.6
	Intermediate professions	95	33.1
	Employees	91	31.7
Renunciation of care utilization before pregnancy			
	No	276	92.0
	Yes	24	8.0
Parity * (children)			
	Mean ± SD (min–max)	1.2 ± 1.0	(0–6)
	0	81	27.7
	1	119	40.8
	≥2	92	31.5
Smoking during pregnancy *			
	No	254	87.3
	Yes	37	12.7
Perceived health (score from 0 to 100)			
	Mean ± SD (min–max)	80.7 ± 17.6	(12–100)
	Median (Q1–Q3)	85	(76–93)
General anxiety (score from 0 to 100)			
	Mean ± SD (min–max)	32.6 ± 24.3	(0–100)
	Median (Q1–Q3)	27	(13–49.5)
Increased general anxiety during pregnancy *			
	No	138	46.3
	Yes	160	53.7

* Missing data; Q1: First Quartile; Q3: Third Quartile; SD: Standard Deviation.

**Table 2:** Women’s knowledge about EDC.

Detailed Knowledge		*N*	%
Ever heard about EDC			
	No	163	54.3
	Yes	137	45.7
Knowledge about EDC score mean ± SD (min–max)		42.9 ± 9.8	(13.5–75.7)
Perceived knowledge about EDC mean ± SD (min–max)		19.0 ± 16.6	(0.0–78.0)
Molecule cited			
	Pesticides	36	26.3
	Bisphenol A	35	25.6
	Parabens	33	24.1
	Phthalates	8	5.8
	Nitrates	2	1.5
	Heavy metals	2	1.5
	Polychlorinated biphenyls	1	0.7
	Alkylphenols	1	0.7
	Phyto-estrogens	1	0.7
	Flame retardants	0	0.0
EDC definition			
	Molecule altering the functioning of the body	250	83.3
	Drug	131	43.7
	Chemical molecule	196	65.3
	Molecule produced by the body	131	43.7
	Natural molecule	120	40.0
	Hormonal molecule	99	33.0
	Bacterium	55	18.3
Source of EDC exposure			
	Cosmetics	274	91.3
	Personal care products	260	86.7
	Prepared dishes	255	85.0
	Tap water	246	82.0
	Cans	241	80.3
	Drug	240	80.0
	Canned food	232	77.3
	Vacuum packed products	204	68.0
	Fresh products	168	56.0
	Bottled water	135	45.0
	Untreated vegetable	84	28.0
Way of EDC exposure			
	Food	296	99.0
	Skin	265	88.6
	Drinking water	264	88.3
	Inhalation	228	76.3
	Blood	155	51.8
Knowledge of plastic packaging resin identification codes			
	No	152	50.7
	Yes	148	49.3

EDC: Endocrine Disrupting Chemicals, SD: Standard Deviation.

**Table 3:** EDC risk assessment towards health issues.

Health Issues		Perceived Severity EDC Risk Assessment “in a General Way”						Perceived Susceptibility EDC Risk Assessment for the Women and Their Child
		Null		Low			High	Mean ± SD
		*n*	%	*n*	%	*n*	%	
Women’s health		0	0.0	87	29.1	210	70.9	53.5 ± 22.1
Children’s health								
	Prematurity	12	4.0	89	29.7	199	66.3	53.0 ± 26.6
	Congenital anomaly	9	3.0	72	24.0	219	73.0	51.7 ± 26.9
	Allergy	14	4.7	105	35.0	181	60.3	54.4 ± 24.1
	Low weight at birth	42	14.0	137	45.7	121	40.3	45.2 ± 25.8
	Fertility disorder in adulthood	29	9.7	86	28.7	185	61.7	49.5 ± 27.6
	Entering puberty at the right time (not too early or too late)	56	18.7	125	41.7	119	39.7	45.5 ± 27.7
	Cancer in adulthood	21	7.0	94	31.3	185	61.7	53.1 ± 26.8
	Overweight or obese as a teenager	71	23.7	134	44.7	95	31.7	40.9 ± 25.2
	Asthma	38	12.7	107	35.7	155	51.7	50.7 ± 25.8
	Immune deficiency	35	11.7	127	42.3	138	46.0	46.9 ± 25.8
	Cognitive disorders	71	23.7	137	45.7	92	30.7	41.3 ± 25.1
	Behavioral disorders	76	25.3	134	44.7	90	30.0	39.1 ± 25.2
	Motor development disorders	62	20.7	127	42.3	111	37.0	41.4 ± 26.8

EDC: Endocrine Disrupting Chemicals; SD: Standard Deviation.

**Table 4:** Predictors of increased situational anxiety after questionnaire.

Characteristics			Women with Increased Anxiety	Probability of Increased Anxiety					
		*n*	*n* (%)	OR	95% CI	*p*	OR ^#^	95% CI	*p*
Age									
	18–25	33	18 (54.5)	Ref			Ref		
	26–35	206	126 (61.2)	1.31	(0.63–2.75)	0.472	1.15	(0.53–2.49)	0.727
	>35	61	42 (68.9)	1.84	(0.77–4.41)	0.171	1.25	(0.49–3.19)	0.634
Perceived health									
	Q1	78	50 (64.1)	Ref			Ref		
	Q2	74	45 (60.8)	0.87	(0.45–1.68)	0.675	0.83	(0.41–1.68)	0.610
	Q3	80	56 (70.0)	1.31	(0.67–2.54)	0.431	1.31	(0.65–2.66)	0.447
	Q4	68	35 (51.5)	0.59	(0.31–1.15)	0.124	0.50	(0.25–1.02)	0.057
Knowledge score on EDC									
	Q1	93	53 (57.0)	Ref			Ref		
	Q2	63	35 (55.6)	0.94	(0.50–1.80)	0.859	1.15	(0.56–2.36)	0.696
	Q3	71	44 (62.0)	1.23	(0.65–2.31)	0.520	1.43	(0.73–2.81)	0.301
	Q4	73	54 (74.0)	2.15	(1.10–4.17)	**0.024**	2.30	(1.12–4.71)	**0.023**
Increased anxiety during pregnancy *										
	No	138	92 (66.7)	Ref			Ref		
	Yes	160	93 (58.1)	0.69	(0.43–1.11)	0.130	0.57	(0.34–0.95)	**0.030**
Parity *									
	0	81	48 (59.3)	Ref			Ref		
	1	119	69 (58.0)	0.95	(0.54–1.68)	0.857	0.97	(0.53–1.77)	0.915
	≥2	92	64 (69.6)	1.57	(0.84–2.94)	0.158	1.81	(0.93–3.55)	0.083

^#^ Adjusted Odds Ratio on age, perceived health, knowledge score of ED, increased anxiety during pregnancy and parity; * Missing data; Q: Quartile; OR: Odds Ratio; CI: Confidence Interval; SD: Standard Deviation.

## Appendix A

**Table A1:** Characteristics of semi-structured individual interviews participants.

Characteristics	Interviews of Pregnant Women
	*N* = 12
Age (years)	
18–24	3
25–29	3
30–34	3
>35	3
Employment status of pregnant women	
Unemployed	2
Artisan, Merchant, Business leader	1
Executive, Intellectual profession	2
Intermediate profession	2
Employed	5
Employment status of the husband or partner	
Unemployed	1
Artisan, Merchant, Business leader	1
Executive, Intellectual profession	4
Intermediate profession	1
Employed	5
Place of residence	
Urban	7
Rural	5
Accommodation type	
House	10
Apartment	2
Primiparity	
No	4
Yes	8

**Table A2:** Characteristics of the focus group participants.

	Gender	Profession	Workplace
1	Female	Midwife (student)	University Hospital
2	Female	Pediatric nurse	French departmental structure responsible for mothers and their children’s protection
3	Female	Prevention psychology (student)	French association involved in health education and promotion
4	Female	Project leader	French health care mutual
5	Female	Workshop organizer	French health care mutual
6	Male	Project leader	French association involved in health education and promotion
7	Male	PhD student	University Hospital
